# Outcomes of Single Anastomosis Duodeno-Ileal Bypass With Sleeve Gastrectomy (SADI-S): A Single Bariatric Center Experience

**DOI:** 10.7759/cureus.76150

**Published:** 2024-12-21

**Authors:** Muhammad Ijlal Haider, Mandeep Kaur, Zohaib Jamal, Qutayba Almerie, Jennifer Darrien, Duncan Stewart, Muhammad Shafiq Javed, David Daniel Kerrigan

**Affiliations:** 1 Bariatric Surgery, Phoenix Health, Chester, GBR; 2 Department of Surgery, Wrightington, Wigan and Leigh NHS Foundation Trust, Wigan, GBR

**Keywords:** bariatric surgery outcomes, duodenal switch, sadi-s, sleeve gastrectomy, weightloss

## Abstract

Introduction

Bariatric surgery is increasingly employed to address the global burden of morbid obesity, with Roux-en-Y gastric bypass (RYGB) representing the predominant procedure. However, some patients, particularly those with extreme obesity (BMI >50 kg/m²), may experience unsatisfactory weight-related outcomes following RYGB. While biliopancreatic diversion with duodenal switch (BPD-DS) offers superior weight reduction for this population, its complexity and associated risks limit its widespread use. Single anastomosis duodeno-ileal bypass with sleeve gastrectomy (SADI-S) was developed as a simpler substitue to BPD-DS, aiming to gain equivalent weight loss with improved safety profile. This analysis examined the efficacy of SADI-S in facilitating weight loss and ameliorating concomitant conditions (type 2 diabetes mellitus, hypertension, and obstructive sleep apnea) at various time points up to nine years post-procedure. Additionally, the study assessed the incidence of nutritional deficiencies following SADI-S.

Material and methods

This retrospective analysis utilized data obtained from patients undergoing SADI-S at a high-volume specialized bariatric centre between January 2016 and December 2022. Inclusion criteria encompassed individuals with high BMI and uncontrolled obesity-related comorbidities who had experienced insufficient weight-related outcomes following a previous bariatric surgery and demonstrated good compliance within a Tier 3 weight management program. Patients were excluded if they were non-compliant, unfit for surgery, or under 18 years old. Participants were followed up quarterly in the first year, every four months in the second year, and yearly thereafter. Nutritional assessments were performed in accordance with British Obesity and Metabolic Surgery Society (BOMSS) guidelines. Relevant data were extracted from patient clinical records and institutional electronic databases and analyzed using Microsoft Excel.

Results

There were 4000+ bariatric procedures conducted during this time. Seventeen patients were deemed as suitable candidates for SADI-S after multidisciplinary team discussion. The surgeries were performed by various surgeons. Most participants were women, with an age range of 23 to 64 years. The median BMI was 63.4 kg/m² (range: 35 kg/m² to 96.2 kg/m²). Mean percentage excess weight loss (%EWL) was 32% (range: 15% to 53%) at six months, 69.6% (range: 34% to 90%) at one year, and 86.2% (range: 43% to 109%) at two years post-procedure. Extended follow-up data (six to nine years) were available for 16 patients, with one patient lost to subsequent assessments. At this stage, the mean %EWL was 76% (range: 62% to 96%). No patients developed protein-energy malnutrition. However, micronutrient deficiencies were observed, with four patients (23.53%) exhibiting zinc deficiency and three (17.65%) presenting with calcium deficiency. All deficiencies were successfully managed with oral supplementation.

Conclusion

SADI-S effectively achieves substantial and sustained weight loss with high comorbidity resolution in individuals with high BMI. While micronutrient deficiencies require proactive management, SADI-S appears safe and yields favourable outcomes comparable to, or exceeding, those reported in larger studies. Long-term weight maintenance is promising. This study contributes valuable real-world evidence supporting the efficacy and safety of SADI-S, though larger studies are needed for further validation.

## Introduction

With obesity posing a significant public health burden worldwide, bariatric surgery is now widely recognized as fundamental treatment modality in the management of severe obesity. Long-term follow-up data consistently affirm its effectiveness in yielding long-term weight loss and enhancing health outcomes in patients with obesity [1,2]. The exponential rise in bariatric procedures reflects the growing recognition of surgical intervention as a conventional treatment for severe obesity [3]. Since its inception in the 1960s, the Roux-en-Y gastric bypass (RYGB) has remained the cornerstone of surgical weight reduction for most bariatric teams, offering substantial weight loss (85% excess weight loss at two years) and significant metabolic benefits, including type 2 diabetes remission [4]. While RYGB is effective, a subset of patients, particularly those with severe obesity (BMI > 50 kg/m²), may experience insufficient weight reduction or weight recidivism over an extended period [5]. 

In this population, biliopancreatic diversion with duodenal switch (BPD-DS), which was proposed in 1987 by DeMeester et al., is recommended based on empirical evidence demonstrating its efficacy in promoting weight reduction and optimizing metabolic function [6]. Despite its potential benefits, BPD-DS remains underutilized globally (<1%) due to increased morbidity, surgical complexity, and the risk of malnutrition [7].

Developed as a simplification of the BPD-DS with the objective of mitigating nutritional deficiencies, the single anastomosis duodeno-ileal bypass with sleeve gastrectomy (SADI-S) procedure, as described by Torres et al. in 2007, is a malabsorptive bariatric option, requiring only one anastomosis [7]. This procedure can be considered a primary intervention for individuals with complex obesity (BMI > 50 kg/m²) and/or metabolic conditions, especially type 2 diabetes mellitus (T2DM) [8]. Additionally, SADI-S may be employed as a revisional surgery in the context of individuals experiencing unsatisfactory outcomes, specifically inadequate weight loss or weight regain, subsequent to bariatric surgery such as sleeve gastrectomy, adjustable gastric banding (AGB), or RYGB [9,10].

This investigation aimed to evaluate the efficacy and safety of SADI-S in inducing weight loss and resolving obesity-related comorbidities, such as type 2 diabetes mellitus, hypertension, and obstructive sleep apnea (OSA). The study assessed outcomes at various intervals post-procedure: six months, one year, two years, and long-term follow-up (six to nine years). Additionally, the study monitored for potential nutritional deficiencies, as malabsorptive procedures can lead to micronutrient deficiencies. Safety was assessed by examining intraoperative mortality, 30-day mortality, and 30-day readmission rates. Efficacy was determined by analyzing percent total weight loss (%TWL) at six, 12, and 24 months, with sustained weight loss defined as ≥50% TWL at the latest follow-up. The impact of SADI-S on comorbidity remission was assessed, defining remission as the absence of clinically significant disease and medication discontinuation or reduction.

## Materials and methods

This single-center retrospective analysis employed a retrospective analytical approach utilizing prospectively collected data to examine outcomes in a cohort of 17 patients who underwent SADI-S at the Phoenix Health Bariatric Referral Center in Chester, United Kingdom between January 2016 and December 2022. Surgical procedures were performed at Alexandra Hospital and Pines Hospital in Manchester, United Kingdom. These 17 patients, selected from a cohort of over 4217 individuals undergoing bariatric procedures during this period, were deemed suitable candidates for SADI-S following a multidisciplinary team (MDT) discussion. The surgical procedures were conducted by various surgeons across the two hospital sites. This study received departmental approval but did not necessitate formal ethical approval, as it involved a retrospective analysis of existing data.

Selection criteria for SADI-S

Patients were selected for SADI-S based on several criteria, including a BMI greater than 35 and the absence of major anesthetic risks, as assessed by an MDT. Additionally, patients were required to demonstrate a clear understanding of the procedure, its potential complications, and the lifelong commitment to medication. Psychological evaluation was conducted, if necessary, to assess the patient's mental health and suitability for surgery. Patients with a history of previous laparotomy were excluded, and those planning pregnancy within the next two years were also ineligible. 

Inclusion criteria

Inclusion criteria for this study encompassed individuals with a high body mass index (BMI) and uncontrolled obesity-related comorbidities who had experienced insufficient weight loss or weight regain following a previous bariatric procedure. Crucially, candidates were required to demonstrate good compliance within a Tier 3 weight management program, as defined by NHS guidelines [11].

Exclusion criteria

Patients were excluded from participation if they exhibited non-compliance with treatment regimens, were deemed unfit for surgical intervention, or were under 18 years of age.

Follow-up protocol

Post-procedure follow-up was conducted quarterly in the first year, every four months in the second year, and annually thereafter. Nutritional assessments, including blood tests, were performed in accordance with the British Obesity and Metabolic Surgery Society (BOMSS) guidelines [12].

Patient demographic and clinical data were extracted from medical records and electronic databases maintained within the ClinicOffice system. A standardized proforma was employed for data collection, and the collated information was subsequently entered into a Microsoft Excel spreadsheet (Microsoft, Redmond, WA, USA) for analysis. The study aimed to assess both the short-term and long-term effects of the procedure. 

## Results

This study included 17 patients undergoing SADI-S. The patient cohort exhibited a median age of 48 years, with the youngest individual being 23 years old and the oldest being 64 years old. The median preoperative BMI was 63.4 kg/m², indicating a high degree of obesity within the study population. BMI values ranged from 35 kg/m² to 96.2 kg/m², reflecting variability in the severity of obesity among participants. The majority of patients were female (n=10, 58.82%), with a smaller proportion being male (n=7, 41.18%). Table 1 provides a descriptive analysis of the study population's baseline characteristics. 

**Table 1:** Characteristics of study population Preoperative BMI represented as kg/m². SADI-S: single anastomosis duodeno-ileal bypass with sleeve gastrectomy; RYGB: Roux-en-Y gastric bypass

Sr. No	Age at the time of surgery (years)	Gender	Pre- Operative BMI (kg/m²)	Final Operation Performed	Length Of Stay (Nights)	Readmission Within 30 Days
1	49	Male	96.2	SADI-S (2-Stage- Sleeve)	2	Yes
2	28	Male	66.5	SADI-S	2	No
3	26	Male	68.4	SADI-S	2	No
4	23	Male	67.7	SADI-S	3	No
5	49	Male	70	SADI-S	3	No
6	64	Female	64	SADI-S	2	No
7	47	Male	55	SADI-S	2	No
8	49	Female	57	SADI-S	2	No
9	29	Male	63.4	SADI-S	2	No
10	43	Female	35	SADI-S (2-Stage-RYGB-SADIS)	2	No
11	48	Female	71	SADI-S	2	No
12	57	Female	49	SADI-S (2-Stage-RYGB-SADIS)	2	No
13	56	Female	73	SADI-S (2-Stage- Sleeve-SADIS)	2	No
14	61	Female	44	SADI-S (2-Stage- Sleeve)	2	No
15	53	Female	41.5	SADI-S (2-Stage-Bypass)	2	No
16	37	Female	45	SADI-S	2	No
17	36	Female	63	SADI-S	3	No

There was no intraoperative or 30-day mortality. Within the study cohort, a single patient (5.88%) experienced a postoperative complication characterized by the simultaneous occurrence of pulmonary embolism (PE) and deep vein thrombosis (DVT), necessitating readmission 30 days following the SADI-S procedure.

**Figure 1:**
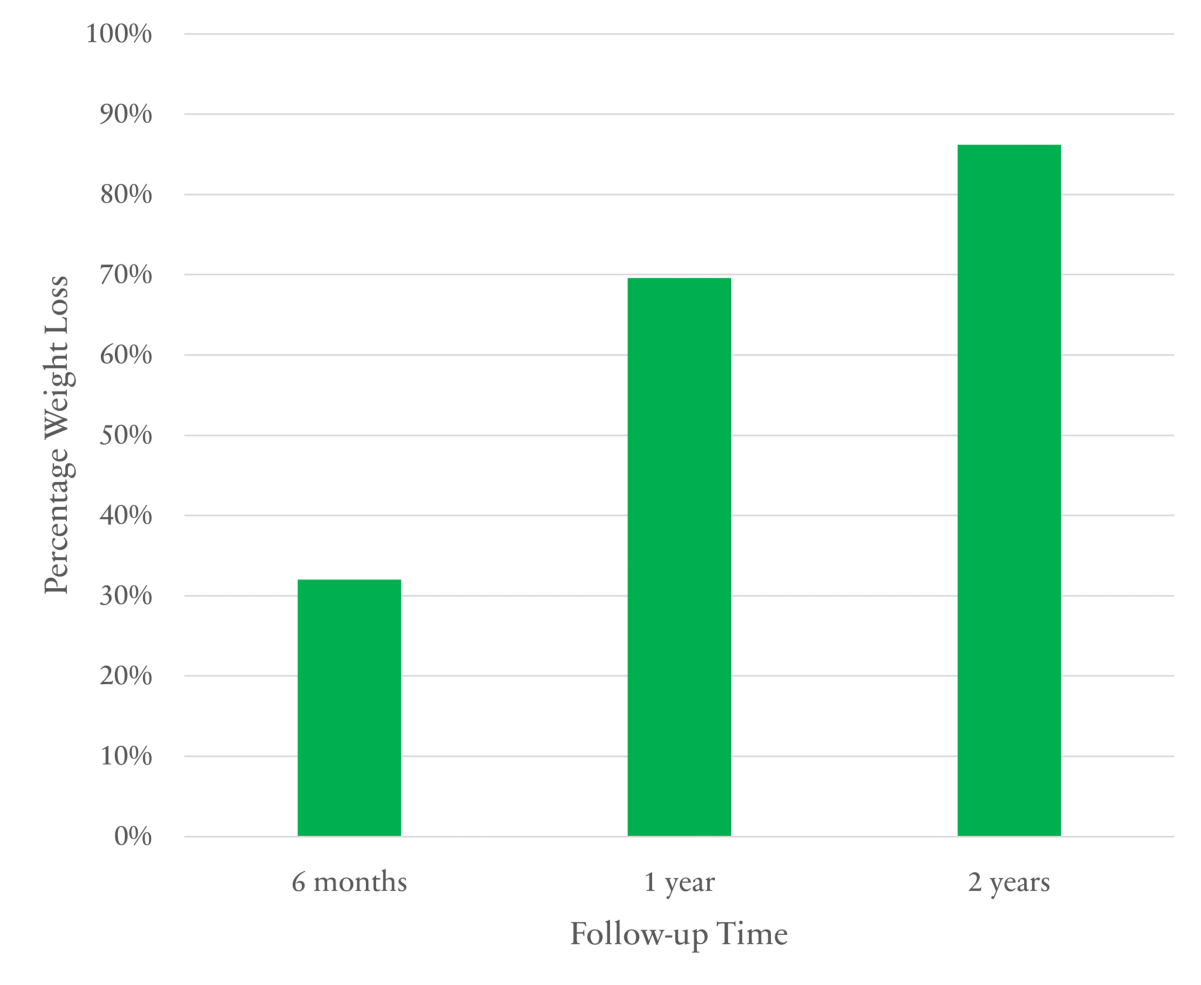
EBWL outcomes after SADI-S at six-month, one-year and two-year follow-ups EBWL: estimated body weight loss; SADI-S: single anastomosis duodeno-ileal bypass with sleeve gastrectomy

The mean percentage of excess weight loss (%EWL) was 32% (range: 15%-53%) at six months, 69.6% (range: 34%-90%) at one year, and 86.2% (range: 43%-109%) at two years as represented in Figure 1.

Adherence to the prescribed follow-up regimen, as outlined in the National Institute for Health and Care Excellence (NICE) guidelines for bariatric surgery, was observed in all but one patient within the study cohort. This individual was lost to follow-up and therefore did not complete the recommended assessments. Table 2 presents a comprehensive overview of weight loss outcomes following the SADI-S procedure. It includes both preoperative and postoperative weights, with postoperative data collected at standardized intervals (six months, one year, and two years) for all patients. Additionally, the table incorporates weight data from variable follow-up periods, capturing longer-term outcomes in a subset of individuals.

**Table 2:** Weight loss outcomes after SADI-S at six-month, one-year, two-year and maximum individual follow-ups. Weight has been represented in kilograms, BMI in kg/m² and EBWL as N(%). EBWL: estimated body weight loss; SADI-S: single anastomosis duodeno-ileal bypass with sleeve gastrectomy

Sr. No	Preoperative Weight (Kilograms)	Pre- Operative BMI (kg/m²)	EBWL in kilograms (%) at 6-Months	EBWL in kilograms (%) at 1-Year	EBWL in kilograms (%) at 2-Years	Maximum Follow up
1	220	96.2	24.45 (15%)	55.42 (34%)	Lost to follow up	No further follow up
2	201	66.5	27.6 (22%)	81.64 (65%)	99.1 (79%)	6-years- 91.7 kg (73% EBWL)
3	218	68.4	33.17 (24%)	92.7 (67%)	117.47 (85%)	6-years- 114.5 kg (83% EBWL)
4	251.5	67.7	37.38 (23%)	146.25 (90%)	169.4 (104%)	6-years- 100.75 kg (62% EBWL)
5	225.5	70	69.94 (48%)	116.5 (80%)	139.45 (96%)	5-years- 139.45 kg (96% EBWL)
6	189	64	28.8 (25%)	101.3 (88%)	114 (99%)	8-years- 114 kg (99% EBWL)
7	176	55	26 (27%)	73 (76%)	79.9 (83%)	9-years- 89.49 kg (93% EBWL)
8	159	57	23.3 (26%)	67.2 (75%)	82.6 (92%)	3-years- 80.64 kg (90% EBWL)
9	206	63.4	40 (32%)	104.7 (83%)	120.9 (96%)	3-years- 121.8 kg (97% EBWL)
10	109	35	7.75 (25%)	25.73 (83%)	26.66 (86%)	3-years- 29.76 kg (96% EBWL)
11	220	71	54.2 (38%)	97.1 (68%)	100 (70%)	3-years- 118 kg (83% EBWL)
12	142	49	23.1 (33%)	53.9 (77%)	58.2 (83%)	3-years- 60.9 kg (87% EBWL)
13	131	73	31.1 (36%)	28.5 (33%)	38 (44%)	No further follow up
14	154	44	35.5 (53%)	54.8 (82%)	63.6 (95%)	No further follow up
15	118	41.5	20.5 (43%)	-	51.9 (109%)	No further follow up
16	235	45	23.2 (22%)	80 (76%)	80 (76%)	3-years-80 kg (76% EBWL)
17	192	63	22.1 (19%)	72.3 (62%)	88.5 (76%)	No further follow up

The SADI-S procedure demonstrated notable efficacy in ameliorating obesity-related comorbidities, with significant improvements observed in hypertension, diabetes mellitus, and obstructive sleep apnea. Complete resolution of OSA occurred in 75% (n=6) of affected individuals. Similarly, hypertension and diabetes mellitus demonstrated comparable resolution rates, with 87.50% (n=7) of patients achieving complete remission from each condition. Table 3 provides a detailed overview of the pre-existing physical and psychological conditions observed within the study population. Furthermore, it presents the outcomes pertaining to the resolution of obesity-related comorbidities following the SADI-S procedure.

**Table 3:** Comorbidity outcomes (six to nine years) following SADI-S PE: pulmonary embolism, OA: osteoarthritis, IBS: irritable bowel syndrome, GORD: gastroesophageal reflux disease, Rh factor: Rhesus factor, HTN: hypertension, SADI-S: single anastomosis duodeno-ileal bypass with sleeve gastrectomy, DM: diabetes mellitus, OCD: obsessive-compulsive disorder, OSA: obstructive sleep apnea, PTSD: post-traumatic stress disorder

Sr. No	Patient Pre-existing Comorbidities	Psychological Issues	OSA	OSA Remission	DM	DM Remission	HTN	HTN Remission
1	Multiple PEs Chronic Bronchitis OA – Knees & Spine	Poor eating pattern	N	-	Y	Y	N	-
2	-	OCD	N	-	N	-	N	-
3	OA, GORD	Anxiety and palpitations	N	-	N	-	N	-
4	GORD, IBS	Complex mental health needs, Laxative abuse, Food phobia, unstable personality disorder	N	-	N	-	N	-
5	Atrial Fibrillation	Depression- weight related	Y	Y	Y	Y	Y	Y
6	-	-	N	-	Y	Y	Y	Y
7	-	-	Y	Y	N	-	Y	N
8	OA, 7 miscarriages- Rhesus Factor negative	Depression, Anxiety, Complex PTSD	Y	N	Y	Y	Y	Y
9	-	-	Y	Y	N	-	N	-
10	-	-	N	-	N	-	Y	Y
11	-	Depression, Anxiety, Agoraphobia, Panic attacks	N	-	N	-	Y	Y
12	Polycystic ovary, Asthma, Arthritis, Lymphedema	Polycystic ovary, Asthma, Arthritis, Lymphedema,	N	-	Y	N	N	-
13	Osteoarthritis, Depression, Asthma	Alcohol abuse, Depression	Y	N	Y	Y	N	-
14	Respiratory arrests because of OSA, OSA resolved with Sleeve, OA- both knees requiring replacement, Arthritis Cholecystectomy	Emotional eating	Y	Y	N	-	Y	Y
15	-	Depression	N	-	N	-	N	-
16	-	-	Y	Y	Y	Y	N	-
17	-	-	Y	Y	Y	Y	Y	Y

While four patients (23.53%) experienced persistent diarrhea in the long term following the SADI-S procedure, it is notable that no instances of severe protein-energy malnutrition requiring hospital admission or intravenous supplementation were observed in this cohort. However, micronutrient deficiencies were observed. Four patients (23.53%) exhibited zinc deficiency and three patients (17.65%) presented with calcium deficiency. Additionally, isolated deficiencies were identified in the following: magnesium (n=1, 5.88%), vitamin B (n=2, 11.76%), and vitamin D (n=1, 5.88%) (Table 4). All instances of deficiency were successfully managed through oral supplementation.

**Table 4:** Nutritional outcomes of study population after single anastomosis duodeno-ileal bypass with sleeve gastrectomy (SADI-S)

Sr. No	Diarrhea or Steatorrhea	Nutritional Deficiency
1	No	Hypocalcaemia, Osteopenia, Vitamin B12, Zinc
2	No	None
3	No	Iron, Hypoalbuminemia, Vitamin D
4	Yes (severe preoperatively, exacerbated postoperatively)	Hypocalcaemia, Selenium
5	Yes (minimal)	Hypocalcaemia, Selenium, Zinc, Vitamin B12
6	No	None
7	No	None
8	Constipation on and off	Selenium
9	Yes (2-3 times a day)	None
10	No	None
11	No	None
12	No	Not documented
13	No	Not documented
14	Yes (minimal)	Magnesium, Zinc, Omega
15	No	None
16	No	None
17	No	None

## Discussion

Introduced in 2007, SADI-S procedure modifies the BPD-DS by replacing the Roux-en-Y reconstruction with a Billroth II-type duodeno-ileal anastomosis [7,13]. A critical aspect of the single-loop reconstruction inherent to SADI-S is the determination of optimal common channel length. While initial implementations of the procedure employed a shorter channel, accumulating surgical experience and broader adoption of SADI-S have led to an increase in the preferred length [7,13]. This adjustment is supported by evidence suggesting that longer channels mitigate long-term nutritional deficiencies and malabsorption [13]. Moreover, the elimination of an anastomosis in SADI-S confers the advantages of reduced operative time, shorter anesthesia exposure, and a lower incidence of post-operative complications [13,14]. Emerging long-term data underscore the efficacy of SADI-S in the management of morbid obesity and associated comorbidities, thereby generating considerable interest among prominent international surgical organizations such as American Society for Metabolic and Bariatric Surgery (ASMBS) and International Federation for the Surgery of Obesity and Metabolic Disorders (IFSO) [15,16]. Accumulating evidence suggests that SADI-S yields favorable outcomes in addressing the complex interplay of morbid obesity and its related health complications [17,18].

In this study, we present a retrospective analysis of outcomes in 17 patients who underwent SADI-S at a bariatric center in the UK over a seven-year period. The analysis focuses on postoperative weight loss, the incidence and severity of nutritional deficiencies, and the occurrence of complications.

Our data showed that patients with a minimum of two years of follow-up demonstrated a median %EWL of 86.2%. These findings align with, and in some instances slightly exceed, those reported in the existing literature, further reinforcing the established efficacy of SADI-S in achieving substantial weight loss [19,20]. At 24 months post-surgery, the reported %EWL ranged from 73.91% to 85.96%. This indicates that, on average, patients who underwent SADI-S achieved substantial and sustained weight reduction two years after the procedure [19-22]. The variability in %EWL likely reflects individual factors such as adherence to dietary recommendations, lifestyle modifications, and variations in metabolic response to the surgery.

The efficacy of SADI-S in achieving remission of obesity-related comorbidities has been extensively documented within the current body of literature. Our findings revealed notably high resolution rates for diabetes mellitus (87.50%), exceeding the 63.7% to 78.6% range reported in previous studies [20,23]. Similarly, hypertension resolution in our cohort reached 87.50%, surpassing the 42.4% to 66.4% reported elsewhere [20,23-25]. Furthermore, complete resolution of OSA was observed in 75% of affected individuals, a rate that compares favorably to the 47.4% to 59.6% resolution documented in larger studies [20,24-26]. While these findings are promising and suggest that SADI-S may be particularly effective in mitigating these specific comorbidities, it is important to acknowledge the limitations inherent in this study, notably the relatively limited sample size.

Postoperative complications were relatively infrequent in this study. A single patient (5.88%) experienced a combined PE and DVT requiring readmission 30 days after the SADI-S procedure. Notably, there were no instances of anastomotic leakage, a severe complication that has been reported in other studies [27,28]. Our findings are consistent with those of Pennestrì et al., who observed a 3.3% rate of early postoperative complications, with 0.8% requiring readmission and 1.7% necessitating reoperation [29]. The absence of anastomotic leakages in our cohort is particularly encouraging, as leakages represent a significant source of morbidity and mortality following bariatric surgery. This positive outcome may be attributed to meticulous surgical technique and adherence to established best practices. However, it is again very crucial to acknowledge the limitations of our study's sample size when interpreting these results.

While SADI-S effectively addresses excess weight and comorbidities, it also carries the risk of nutritional deficiencies. In our study, 23.53% of patients experienced persistent diarrhea, a common side effect of malabsorptive procedures. Although no cases of severe protein-energy malnutrition requiring hospitalization were observed, micronutrient deficiencies were prevalent. Zinc (23.53%) and calcium (17.65%) deficiencies were most common, followed by isolated deficiencies in magnesium, vitamin B, and vitamin D. These findings corroborate previous reports [20,22-24,30] that highlight the vulnerability to nutritional deficiencies following SADI-S, particularly deficiencies in vitamin D, calcium, and various vitamins. Importantly, all deficiencies in our cohort were successfully managed with oral supplementation, emphasizing the importance of vigilant monitoring and proactive management of nutritional status in this patient population.

We would also like to comment on the role of choice of patients. Patient selection for the SADI-S procedure was rigorous and involved a comprehensive MDT discussion. Input from dieticians, pharmacists, nurses, and psychologists was mandatory before offering SADI-S to any patient. This ensured a holistic assessment of the patient's suitability for the procedure, considering not only their physical health but also their nutritional status, medication requirements, and psychological readiness. Furthermore, patients received thorough counseling regarding the lifelong need for nutritional monitoring and supplementation, and informed consent was obtained prior to the surgical intervention.

This study employed a descriptive methodology to examine the outcomes of the SADI-S procedure. The study cohort was limited to 17 patients. Consequently, the study focused on characterizing the observed clinical outcomes and providing descriptive statistics. Due to the small sample size and incomplete long-term follow-up data, inferential statistical analysis, including the calculation of p-values, was not conducted. This study is also limited by its retrospective, single-center design. These factors may limit the generalizability of the findings. The small sample size may underrepresent the full range of outcomes and complications associated with the SADI-S procedure. Furthermore, conducting the study at a single center introduces potential selection bias. The outcomes observed in this study may not accurately reflect those achievable in other clinical settings. The retrospective design, reliant on existing medical records, carries a risk of information bias due to potential inaccuracies or incomplete data. Additionally, the relatively short follow-up duration for some patients may not fully elucidate the long-term efficacy and safety profile of SADI-S. Larger, prospective, multicenter studies are necessary to overcome these limitations and provide more robust evidence regarding the long-term outcomes of the procedure.

## Conclusions

This study establishes SADI-S as a promising procedure that can be done to achieve substantial and sustained weight loss in patients with elevated BMI and obesity-related comorbidities. Our results indicate that SADI-S yields significant weight reduction and high rates of comorbidity resolution, comparable to or exceeding those reported in larger studies. While the procedure is generally safe and well-tolerated, it is associated with a risk of micronutrient deficiencies, highlighting the need for close monitoring and proactive management of nutritional status. Notwithstanding the inherent limitations of this study, namely the restricted sample size and single-center design, the findings offer valuable real-world evidence that further substantiates the expanding body of evidence for the weight loss outcomes and complication rates associated with SADI-S. Larger, multicenter studies are warranted to further validate these observations and establish the long-term durability of SADI-S outcomes.
